# Our Duty to Our Profession

**Published:** 1887-01

**Authors:** E. P. Beadles

**Affiliations:** Danville, Va.


AMERICAN JOURNAL

—OF—

DENTAL SCIENCE.

VOL. XX. THIRD SERIES—JANUARY, 1887. No. 9.

ARTICLE I.

### OUR DUTY TO OUR PROFESSION.

BY E. P. BEADLES, D. D. S., DANVILLE, VA.

[Read before the Virginia Dental Association, August, 1886.]

If you were to question me as to why I am practicing dentistry, my first reason, high over all, would be because I love it. Indeed, if this were not the case, I should not have the honor, nor should I deserve the honor, of calling you brethren. I am sure you will agree with me when I say that if I had no love for your profession, but merely entered your ranks for what I could make out of you, I would be utterly unworthy of your notice or recognition. And yet, my friends, we have such men in our profession, men unprincipled to the last degree, men caring nothing for the elevation or success of dentistry, but would, with no hesitation, sacrifice its honor and dignity for the pittance which might be made by so doing. Can any profession succeed or attain to high degree if such men predominate? They pull down as fast as others build up—they work disgrace while a few do honor. These men, with the ignorant and incompetent, have been holding us at the bottom, despite the Herculean efforts of the few educated and truly professional, to raise dentistry to the level to which it truly belongs. But we may thank heaven that a brighter day is dawning—men of culture and high attainments are amongst us, men who study dentistry as a scientific profession, and will eventually place it far beyond the reach of many who have heretofore disgraced it. Any business or profession is just what the men in it choose to make it, and it is a well-known principle that men rarely make a real success of a thing unless they are interested in what they are doing. Can a painter successfully reproduce the picture upon the canvass unless his heart be in the work? Can a sculptor be successful in chiseling out what is truthfully to represent nature unless he is eminently devoted to his work? We answer, No. Then how much more important for a physician or dentist who would successfully treat a diseased part of the human system, where it is especially necessary that he should be sympathetic and kind, adapting himself to all the various temperaments which may come under his hand, that he should be devotedly attached to his work. This is the true key to success, and when we shall be able to fill our ranks with educated men who will love their profession, then indeed will we have a revolution, and dentistry will take its place among the most learned. I might say here that my advice to any young man just beginning would be this: "If your motives are not based upon devotion and high principles, so far as your real success is concerned, or the good you will do your profession, you had better seek something else, for you will never attain to that degree of perfection worthy to be expressed by the word success." We want men who will render themselves ornaments and benefactors. We must be able to look beyond mere personal interests if we make the future of dentistry what many of us wish to see it. We should not wrap ourselves in a cloak of contentment and think that "we are well enough," leaving the real burden to be born by the few devoted ones. We should feel that whatever concerns our profession concerns us, and ever be watchful and jealous of all its interests. Let us be true men, true to ourselves and true to our work. We have a mission to perform—let us not shrink from it. The public is gradually learning to appreciate us and our work, and by making ourselves skillful, with intelligence to back us, our future success as a profession, and as a *scientific* profession, is assured. But to accomplish the end, we must ever be devoted.

Our predecessors have done nobly, as is proven by the enormous strides that have been taken during the last two or three decades. And how has this been accomplished? By hard, unceasing toil, backed by an earnest devotion that never wearied. We have many advantages over these men, though they have done so much. We have good colleges which afford us fine opportunities; improved instruments, new methods of treatment, and it will indeed be our own fault if we do not leave dentistry upon a higher plane than that upon which we found it. Surely this is our duty to our profession. Let us be up and doing. Why should our own Virginia Association be behind any in the land? We have brains here. We have thinkers among us. If we lack anything, it is that self-sacrificing devotion which is so essential to success. But there are some who are far from being deficient even in this respect, as is abundantly proven by the time and thought, and careful fostering spent in the interests of this Association, when there seemed little to encourage its future existence. We honor these men, and our hearts go out in gratitude to them for what they have preserved for us to enjoy, and as they pass into the "sear and yellow leaf," may they look back upon a well-spent life, feeling that their labors and devotion are being appreciated by those who follow them.

As said above, it is our desire to place dentistry upon a level with any of the sciences of the day. This is our work for the future. We owe it to the profession: therefore it becomes our duty. Legislation has stepped in to help us. Many of the charlatans which have heretofore been as millstones about our necks, will have to step down and out. But we must not rest satisfied here. Our law must be amended so that all, whether graduates or non-graduates, shall be compelled to come before our Examining Board. This will place a check upon the colleges that seem to grind out graduates by patented processes. By all means should our colleges require preliminary examinations from students before allowing them to enter.

We can never succeed so long as we have our profession filled with uncultured, uneducated men, and we must rid ourselves of them; our profession has been abused and maligned long enough on account of such. Heretofore we have been allowing any and everybody who could raise money enough to buy a license and a few instruments, to come in, and welcome, and we, as a profession, have suffered for it. It is our duty that we use every means in our power to keep out such men, certainly, if we value our honor. If the colleges will not help us, we must help ourselves. This subject of education is *the* question for us to consider. The future of dentistry is depending upon it, and every day we delay, matters are becoming worse. It is not possible for us to raise ourselves to a desired standard without education. Our friends of the medical fraternity are unfortunate in this respect also. The time was when a man did not think of studying medicine until he had thoroughly prepared himself by a collegiate course; but now men are graduated M. D.'s who cannot write English, saying nothing of not being able to read a word of the Latin diploma which is given them. Our future depends upon the kind of men we graduate and send out. It may not be a man's fault that he has not been able to obtain an education; but this is no reason why our profession should be imposed upon, and be made to suffer. When we shall send out none but proficient men, then it will be that the public will have a proper appreciation of us and our skill. You cannot expect a man to seek advice from one more ignorant than himself. I repeat, this, and this alone, is what is keeping us down, and will keep us down until we right the wrong. I hope our Board of Examiners will consider a deficiency in a man's English education sufficient grounds upon which to reject. We don't want men who can barely read and write, and the sooner they know it the better. I am in favor of rigidness, even compared with the extreme laxity of the past. In the future we will have fewer men imposing upon the public their circulars proposing to "Extract teeth without pain." "Excellent and durable sets of teeth for six dollars." "Gold fillings guaranteed for one dollar." The intelligent public soon finds out these to be humbugs, and are often inclined to judge us, as a profession, by them. By countenancing such we reduce our profession to a trade, and there is absolutely nothing left to which a true man may attach himself. It is our duty to cultivate a love for science for its own sake, ever delving, that we may add a mite to our store of knowledge, to be used for the benefit of those who entrust themselves to our care, and ever endeavor to develop an intelligent appreciation of the resources of science and art which are used to lessen the ills of humanity. Let us forget self, and by earnest devotion ever push upward our profession, always considering its interests, honor and glory. We should emulate our immediate predecessors, carry on the work they have commenced, and never weary until we shall be prouder still of the achievements of this noble science, never flagging until we shall have placed it so high that *any* may feel proud to be numbered among the intelligent and skillful of its members. We have a future before us, a bright future. Let us look to it. The sun of our success is just below the horizon. Its rays can already be seen. Ere long it will come up in all its glory, and we will feel that the old maxim has proven itself true, that "labor has its reward."

I would say, in conclusion, that my heart is with this Association, and my great desire is to see its membership greatly increase, to see each man with his shoulder to the wheel, never shirking or neglecting a single duty, always ready to do anything that will forward its interests, thereby promoting the interests of the profession. We have only to lay hold of the opportunities offered us, going forward earnestly, and we shall not know the meaning of the word fail. Love for our work is the one thing needful. If we have this, all else will follow. Pleasure and happiness even is then secured, but without it we are failures. This is our great duty to our profession. In the language of a well-known professor to his graduating class, I would say, "Hold fast to this love, if you would not sink from weariness in your earlier years, or find the fruits of success so much dross in your old age."—*Southern Dental Journal.*

